# The Road Performance, VOCs Emission Behavior, and Emission Mechanism of Rubber Powder/SBS-Modified Asphalt

**DOI:** 10.3390/ma18235260

**Published:** 2025-11-21

**Authors:** Xuyan Song, Shengsen Li, Menghao Wang, Pengcheng Shi, Fucheng Guo, Ziyang Zhang

**Affiliations:** 1 School of Civil Engineering, Suzhou University of Science and Technology, Suzhou 215011, China; xysong1632@usts.edu.cn (X.S.); 2313031127@post.usts.edu.cn (S.L.); 1506@mail.usts.edu.cn (P.S.); 2 School of Civil Engineering, Lanzhou Jiaotong University, Lanzhou 730070, China; fcguo@chd.edu.cn; 3 School of Civil Engineering, Southwest Jiaotong University, Chengdu 610031, China

**Keywords:** waste rubber powder, modified asphalt, road performance, volatile organic compounds, emission mechanism

## Abstract

To reduce volatile organic compounds (VOCs) emissions from rubber powder/SBS modified asphalt under high temperatures, the key road performance characteristics of asphalt modified with different proportions of rubber powder/SBS were investigated. The optimal rubber powder proportion was determined. The VOCs emission behavior of rubber powder/SBS modified asphalt before and after desulfurization was compared and analyzed using headspace gas chromatography–mass spectrometry (GC-MS). The VOCs emission mechanisms were revealed through microstructural testing. The results showed that the rubber powder/SBS modified asphalt exhibited good road performance with the optimal rubber powder content of 15%. As more rubber powder was added, the total VOCs emissions increased. Desulfurized rubber powder/SBS modified asphalt demonstrated superior performance in controlling harmful VOCs emissions. Undesulfurized rubber powder/SBS modified asphalt released more complex and toxic components compared to desulfurized rubber powder/SBS modified asphalt. The content of xylene was significantly higher than desulfurized rubber powder. Infrared spectroscopy analysis further validated the GC-MS results. Consistency in functional group changes was shown by both methods. Scanning electron microscopy revealed that the original cross-linked network of the adhesive powder was disrupted by desulfurization treatment. Interfacial activity and dispersion of the particles were enhanced. This led to the establishment of a more stable structural system and a reduction in VOCs emissions. Therefore, road performance was ensured, and VOCs emissions were significantly reduced by desulfurized rubber powder/SBS modified asphalt. This expands the utilization rate of waste rubber powder and reduces its environmental pollution.

## 1. Introduction

With the rapid development of industry, the disposal of waste rubber presents serious challenges. Given the current global trend toward promoting a green circular economy, processing waste rubber into rubber powder for resource recycling shows great development potential [1]. More than 30 years of design and construction experience have been accumulated in China’s expressway construction, and a relatively complete highway transportation network has been established. However, due to the need for maintenance during use, the demand for high-performance asphalt has increased dramatically [2]. Rubber powder/SBS modified asphalt is primarily composed of base asphalt, rubber powder, SBS modifier, and stabilizers. Due to the advantages of both rubber powder and SBS as modifiers, the road performance of asphalt has been greatly improved [3,4,5]. Jatoi et al. [6] also pointed out that the incorporation of polymers like SBS and rubber into asphalt can significantly enhance the mechanical properties of asphalt mixtures. It was also noted that this approach improves pavement durability and reduces lifecycle maintenance costs, which is beneficial for sustainable development. Therefore, rubber powder/SBS-composite-modified asphalt has been widely used in road construction.

However, many environmental issues regarding the use of rubber powder/SBS-modified asphalt under high-temperature conditions have not been resolved. Li et al. [7] found that significant amounts of VOCs are released by asphalt samples when temperatures exceed 150 °C; Gong et al. [8] found that VOCs were released by asphalt pavements during use, posing environmental and health risks. A thorough understanding of asphalt VOCs emissions is considered essential for the development of targeted reduction measures. It has been shown in studies that VOCs emitted from asphalt can affect human growth and development [9], impact the nervous system [10], cause organ irritation, breathing difficulties, and even certain cancers [11]. From an environmental perspective, land and water sources can be contaminated by particulate matter generated by VOCs [12] and the atmospheric environment can be affected [13]. Compared with traditional asphalt, more complex and higher concentrations of VOCs emissions are exhibited by rubber powder/SBS asphalt. The rubber powder in unsulfurized rubber powder/SBS-modified asphalt (UDRA) is mostly derived from waste rubber tires, and the main component of tire rubber is vulcanized polymer. Under high-temperature conditions, sulfur-containing fumes and volatile organic compounds are easily released during the modification process [14] and large amounts of VOCs and sulfur compounds were generated [15,16,17]. These substances are not only the main components of rubber exhaust gases but also the primary sources of malodorous gases, and this is why rubber exhaust gases generally have a strong and pungent odor. The pollutants contained in it pose serious hazards to human health and the ecological environment, and they are likely to cause respiratory, blood, nervous system, and central nervous system diseases in humans [10]. Li et al. [11] found that VOCs emissions from rubber asphalt in the early stages originate from the rubber, transitioning to petroleum asphalt in the later stages; Wang et al. [18] demonstrated that increasing the rubber powder content and heating temperature in rubber asphalt significantly increased VOCs emissions. A large number of VOCs, such as benzene, toluene, ethylbenzene, xylene, and sulfur compounds, were identified in rubber asphalt emissions by Gągol et al. [19].

GC-MS is widely used for the detection of VOCs in asphalt materials due to its high detection sensitivity. Compared to Fourier Transform Infrared Spectroscopy (FTIR), specific gas products can be identified by GC-MS rather than a group of compounds with the same functional group characteristics [20]. The advantage of the GC-MS analysis method is that compounds in complex mixtures can be separated through chromatography, and then analyzed by mass spectrometry ionization, and standard spectral libraries are used to accurately identify unknown compounds. Yang et al. [21] used pyrolysis GC-MS to study the emissions of carbon dioxide and VOCs in asphalt fumes. Qin et al. [22] used GC-MS to track the evolution of light components in recycled asphalt. Tang et al. [23] dissolved samples collected under different conditions in carbon disulfide and used GC-MS to quantify the emissions.

Currently, the primary methods for reducing emissions from rubber powder/SBS-modified asphalt are divided into two categories: the addition of emission reduction agents and mechanical desulfurization. Wang et al. [24] and E et al. [25] reduced VOCs emissions from road asphalt at high temperatures by preparing asphalt emission reduction agents. Lin et al. [26] reduced VOCs emissions from rubber asphalt by using inhibitors. Jia et al. [27] absorbed pollutants from asphalt fumes using molecular sieves to lower the preparation temperature. However, the performance of asphalt in road applications may be affected by the addition of emission reduction agents. Mechanical desulfurization has the advantages of simple equipment, high production efficiency, and low cost [28]. The C-S bonds in the gum powder can be effectively broken, reducing the generation of volatile organic compounds under high-temperature conditions.

To reduce VOCs emissions from rubber powder/SBS-modified asphalt under high temperatures, we investigated the key road performance characteristics of asphalt modified with different proportions of rubber powder/SBS and determined the optimal rubber powder proportion. The VOCs emission behavior of rubber powder/SBS-modified asphalt before and after desulfurization was compared and analyzed using GC-MS, with infrared spectroscopy analysis further validating the GC-MS results. Finally, the VOCs emission mechanisms were revealed through microstructural testing.

## 2. Methodology

**Figure 1:**
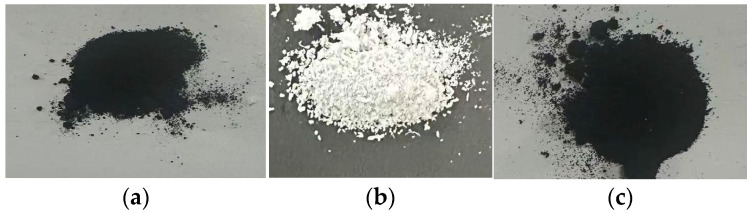
Experimental raw materials. (**a**) Desulfurized rubber powder; (**b**) SBS; (**c**) Undevulcanized rubber powder.

Figure 1 lists the raw materials used in this study, from left to right: devulcanized rubber powder, SBS modifier, and undevulcanized rubber powder.

### 2.1. Raw Material

Double Dragon 70# base asphalt from Suzhou Sanchuang Road Engineering Company was employed. The basic indicators of the base asphalt were determined according to the test requirements specified in ASTM International, including ASTM D6114 [29], ASTM D5603 [30], and ASTM D5755 [31], as shown in Table 1.

The unsulfurized rubber powder was obtained directly by grinding used tires. The desulfurized rubber powder (80 mesh) was produced using desulfurized rubber powder processed by the screw extrusion method as the raw material for research. The rubber powder was mixed with the desulfurizing agent under high shear force and extruded, allowing the desulfurizing agent to penetrate the interior of the rubber powder. Under the action of heat and oxygen, a chemical reaction occurred, breaking the molecular chains of the rubber powder and generating new active groups, which activated and regenerated the rubber powder [32]. The technical specifications were listed in Table 2. The addition ratio of the SBS modifier was 4%. The technical specifications are shown in Table 3.

Desulfurized rubber powder is abbreviated as DR, unsulfurized rubber powder is abbreviated as UDR, desulfurized rubber powder/SBS-modified asphalt is abbreviated as DR/SBSA, and unsulfurized rubber powder/SBS-modified asphalt is abbreviated as UDR/SBSA. SBS-modified asphalt is abbreviated as SBSA.

Preparation of SBSA: The base asphalt was heated to 145 °C. An amount of 4% SBS was added, and the mixture was sheared for approximately 40 min by a high-speed emulsifying shear mixer (Shanghai Aido BME100LT, Shanghai, China). Then, 0.3% stabilizer was added, stirred for 1 min, and cured at 175 °C for 1 h.

Preparation of DR/SBSA: The base asphalt was heated to 145 °C. Devulcanized rubber powder at 10%, 15%, and 20% by weight was added, and the mixture was thoroughly mixed for approximately 5 min to ensure uniform dispersion of the rubber powder in the base asphalt. Under 4000 rpm and 170–175 °C, 4% SBS was added, and the mixture was sheared for approximately 40 min by a high-speed emulsifying shear mixer (Shanghai Aido BME100LT). Then, 0.3% stabilizer was added, stirred for 1 min, and cured at 175 °C for 1 h.

Preparation of UDR/SBSA: The base asphalt was heated to 145 °C. Undevulcanized rubber powder at 10%, 15%, and 20% by weight was added, and the mixture was thoroughly mixed for approximately 5 min to ensure uniform dispersion of the rubber powder in the base asphalt. Under 4000 rpm and 170–175 °C, 4% SBS was added, and the mixture was sheared for approximately 40 min by a high-speed emulsifying shear mixer (Shanghai Aido BME100LT). Then, 0.3% stabilizer was added, stirred for 1 min, and cured at 175 °C for 1 h.

### 2.2. Test Methods

Table 4 presents the tests conducted on all asphalt samples in this study.

#### 2.2.1. Basic Performance Test

The test methods were conducted following the testing standards specified by ASTM International. All tests in this study were conducted in triplicate, and the reported values are the average of three independent measurements. The experimental errors comply with the requirements of the relevant standards, according to ASTM D6114 [29], ASTM D5603 [30], and ASTM D5755 [31].

Needle penetration: The Wuxi South SYD-2801F fully automatic needle penetration tester produced by Wuxi Huanan Experimental Instrument Co., Ltd. (Wuxi, China) was used. Penetration was determined by the depth to which a standard needle was vertically inserted into the asphalt sample under specified load, temperature, and time conditions. The unit of measurement was 1/10 mm.

Extensibility: The Wuxi Petroleum LYY-10A-1 Extensibility Tester (Wuxi, China) produced by Wuxi Petroleum Instrument Equipment Co., Ltd. was used. The test temperature was 5 °C, and the stretching speed was 5 cm/min. First, the asphalt sample was poured into an octagonal mold. The sample was cooled at room temperature for 1.5 h, then shaved flat along the mold surface using a knife heated by an alcohol lamp. The asphalt and mold were placed in water at 5 °C and cooled for 1 h. Finally, the mold was removed, and the asphalt sample was taken out. It was then placed on the elongation tester to conduct a tensile test and determine the elongation value of the sample.

Softening point: The instrument used was the SYD-2806F produced by Wuxi Huanan Experimental Instrument Co., Ltd. (Wuxi, China). During the test, the water bath temperature was controlled to increase at a rate of 5 °C per minute. First, the asphalt sample was poured into the sample ring until it slightly exceeded the ring surface. After cooling at room temperature for 0.5 h, the sample was scraped along the mold surface to ensure it was flush with the ring surface. The sample, mold, steel ball, and metal support were placed in a water bath maintained at 5 °C for at least 15 min. Then, according to the specified method, the sample was placed in the test container for heating. The temperature was recorded when the steel ball sunk to the specified distance from the asphalt.

Rolling thin-film oven test (RTFOT): The 81-PV1632 rolling thin-film oven was used. First, 35 ± 0.5 g of asphalt sample was poured into a clean, dry glass bottle, and the initial mass was recorded. The oven was then preheated to 163 ± 0.5 °C. The air flow was adjusted to 4000 mL/min. The sample was placed in the oven, and the rotation was started. The aging process was timed for 75 min.

#### 2.2.2. Rheological Property Test

The instrument used was the American TA HR10 produced by TA Instruments (Newcastle, DE, USA). The asphalt sample was poured into a silicone sheet to form a chess piece-shaped sample. A 25 mm parallel plate was used for the test. After the instrument was calibrated, the scraper was heated with an alcohol lamp to remove excess asphalt. The temperature was scanned from 58 °C to 88 °C with a temperature gradient of 6 °C during the test.

#### 2.2.3. Volatile Organic Compound Test

The test was conducted using the Thermo Fisher Scientific ISQ 7000 single quadrupole gas chromatography–mass spectrometry (GC-MS) instrument produced by Guoyue Trading (Shanghai) Co., Ltd. (Shanghai, China). First, the samples were processed and injected into headspace vials, then heated to promote the evaporation of volatile substances. The headspace sampler automatically extracted gases from the samples at a set temperature and time, which were subsequently separated by gas chromatography and detected by mass spectrometry. Unknown substances were identified by comparing their mass-to-charge ratio (m/z) distributions and relative abundances with reference spectra in the database, where match factors (MF) exceeding 90% served as the reliable identification threshold.

#### 2.2.4. Fourier Transform Infrared Spectroscopy (FTIR) Test

A Thermo Fisher IS5 infrared spectrometer produced by Shanghai Kerui Instrument Technology Co., Ltd. (Shanghai, China) was used. Asphalt samples and trichloroethylene were mixed in a 1:10 ratio to form a solution. One drop of the solution was placed on a potassium bromide (KBr) substrate using a dropper, and the solvent was allowed to evaporate naturally to form a uniform asphalt film. The film should be bubble-free and of consistent thickness. The KBr plate with the asphalt film was then placed in the sample chamber of the infrared spectrometer, and the spectrum was recorded.

#### 2.2.5. Scanning Electron Microscopy (SEM) Test

Czech TESCAN MIRA LMS produced by TESCAN (Shanghai) company (Shanghai, China): The asphalt sample was rapidly frozen in liquid nitrogen and then transferred to the SEM cryo-stage to prevent shrinkage or structural deformation caused by drying. A small sample (diameter ≤ 1 cm, height ≤ 5 mm) was taken and directly adhered to the conductive adhesive. The Quorum SC7620 sputtering coating instrument produced by Mecano Technology Co., Ltd. (Hong Kong, China) was used to deposit platinum for 45 s at a current of 10 mA. This step ensures uniform coating deposition across the sample surface, effectively mitigating charge accumulation effects. Subsequently, the sample morphology was captured. During morphology imaging, the acceleration voltage was set to 3 kV.

## 3. Results

### 3.1. Road Performance

#### 3.1.1. Basic Performance

**Figure 2:**
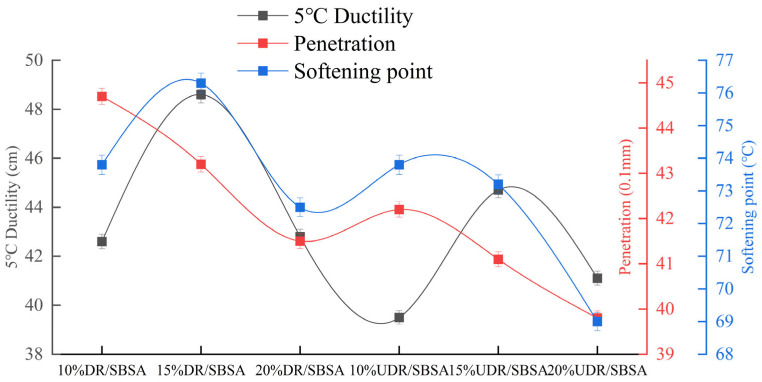
The basic performance of UDR/SBSA and DR/SBSA.

Penetration, ductility, and softening point are used to reflect the high- and low-temperature performance of asphalt, as well as its softness at normal temperatures. The results are shown in Figure 2.

As shown in Figure 2, the penetration of DR/SBSA initially increases and then decreases. This change indicates that a high DR content increases the amount of free rubber powder in the asphalt, and the network structure formed by SBS cannot be effectively filled. This affects swelling and dispersion, accelerating the hardening process of the asphalt. In comparison, the effect of UDR/SBSA is minimal, and the penetration remains high. The elongation of both rubber powder-modified asphalts initially increased and then decreased. An appropriate amount of rubber powder enhances the asphalt’s resistance to deformation. However, when the content exceeds a certain threshold, excessive rubber powder hardens the asphalt. Free rubber powder agglomerates, leading to stress concentration during stretching, which reduces elongation performance [33]. This is consistent with the findings of Huang et al. [34]. They observed that adding excessive rubber powder does not significantly improve the physical properties of asphalt. Rubber powder particles tend to precipitate, leading to uneven dispersion within the asphalt and a reduction in its physical properties.

Additionally, it can be observed that the ductility of the DR/SBSA is superior to that of the UDR/SBSA. This is because the three-dimensional cross-linked network of vulcanized rubber is retained by the unsulfurized rubber powder, restricting the movement of molecular chains. In asphalt, it exists as rigid particles that, under stress, are easily turned into points of stress concentration, limiting overall extensibility. In contrast, the active chain segments of the desulfurized rubber powder can be physically entangled or mildly grafted with the butadiene in SBS, forming a ‘rubber powder-SBS’ interpenetrating network. This network, with its interface to asphalt blurred, facilitates uniform stress transfer and delays crack propagation. It improves low-temperature elongation performance. The softening point significantly increases with higher content of desulfurized rubber powder, especially at a 15% content level. The rise in softening point indicates enhanced high-temperature stability of the asphalt by desulfurized rubber powder.

#### 3.1.2. Rheological Properties

**Figure 3:**
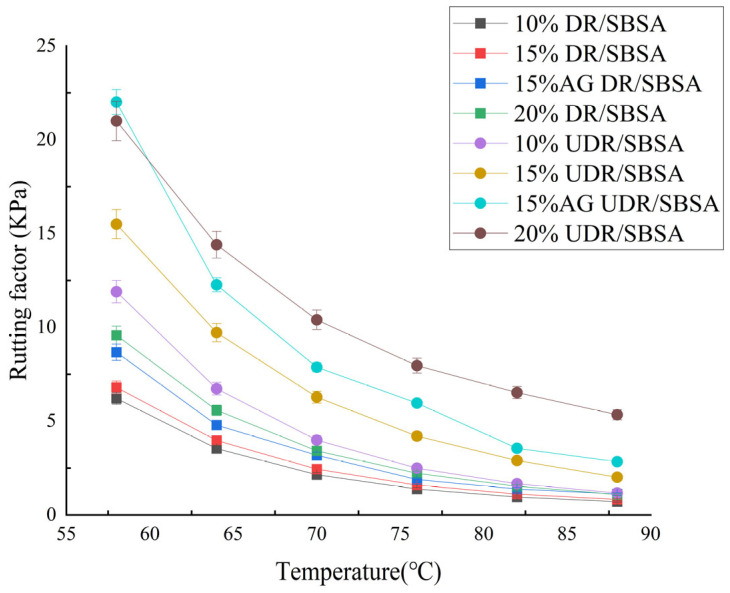
Rheological properties of rubber powder/SBS-modified asphalt.

The rutting factor is used to characterize the ability of asphalt materials to resist permanent deformation at high temperatures. A higher value is indicative of better resistance to rutting and greater resistance to permanent deformation. The relationship between the rutting factors of DR/SBSA and UDR/SBSA and temperature is shown in Figure 3.

As shown in Figure 3, the rutting factors of UDR/SBSA are higher than those of DR/SBSA with different DR contents under different temperatures. The rutting factors of DR/SBSA and UDR/SBSA both increase with the increase in rubber powder content. This indicates that increasing the dosage of rubber powder improves the high-temperature rheological properties of asphalt. A 20% addition of UDR/SBSA results in a significant improvement in the rutting factor. However, excessively high binder powder content leads to asphalt hardening, as shown in Figure 2. Considering low-temperature performance, both DR/SBSA and UDR/SBSA exhibit optimal performance with the rubber powder content of 15%. Therefore, 15% rubber powder/SBS-modified was selected to analyze the emission behavior of volatile organic compounds.

Compared to the unaged modified asphalt, both DR/SBSA and UDR/SBSAshowed an increase in the rutting factor. The rutting factors of DR/SBSA and UDR/SBSA showed a higher increase of 22% and 34%. An increase in rutting factor indicates a deterioration in the rheological properties of asphalt [35]. This phenomenon may be attributed to the alteration of light fractions in the asphalt caused by aging. The changes in these light fractions are the primary reason for VOC emissions. Furthermore, the modification of the light components leads to asphalt hardening and embrittlement, consequently deteriorating its rheological properties.

### 3.2. VOCs Emission Behavior

#### 3.2.1. VOCs Release Characteristic

**Figure 4:**
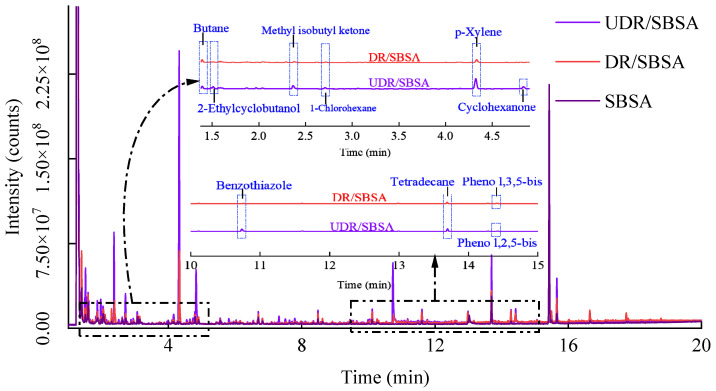
TLC patterns of SBSA, DR/SBSA, and UDR/SBSA.

The release behavior of the VOCs components was preliminarily identified and determined by the total ion chromatogram (TIC), and the retention times of the chromatographic peaks with distinct shapes are 1.5 to 4.5 min or 10 to 15 min, respectively. An enlarged comparison of the chromatographic peaks in the retention time ranges of 1.5 to 4.5 min or 10 to 15 min, as shown in Figure 4.

As shown in Figure 4, the UDR/SBSA exhibits many medium-to-low intensity peaks between 1.5 and 12 min, showing the highest diversity of released components. Strong cracking activity is exhibited by UDRA under high temperatures, resulting in the thermal decomposition of complex organic small molecules and significant VOCs emissions. The VOCs emission characteristic of the DR/SBSA changes significantly. The number of peaks in the spectrum of this group decreased significantly, with most peaks showing low intensities that are primarily concentrated in the 1.5–5 min range. This indicates that the desulfurization treatment effectively disrupts the three-dimensional cross-linked structure of the rubber powder, improves its thermal stability, and moderates the cracking reaction. The released VOCs consisted mainly of low-toxicity, highly volatile components such as branched alkanes and alcohols.

In addition, compared with UDR/SBSA, no potential high-risk pollutants such as cyclohexanone are detected in DR/SBSA, indicating a significant enhancement in environmental release safety. SBSA asphalt exhibits a distinct high-intensity characteristic peak at a retention time of approximately 15 min, identified as 2,4,6-tri-tert-butylphenol. The characteristic peak is detected in all three sample groups, with intensity values decreasing sequentially from SBSA to DR/SBSA to UDR/SBSA. This result indicates that the phenolic compounds predominantly originate from SBS, while their release kinetics are controlled by the rubber powder constituent in the composite matrix. The dense cross-linking network and limited polar sites in the UDR/SBSA structure facilitate the adsorption or embedding of antioxidant macromolecules, consequently suppressing their release and leading to peak attenuation. Following desulfurization treatment, the opened surface morphology of rubber powder promotes component migration and diffusion within SBS, resulting in enhanced relative release efficiency.

From Figure 4, the retention times of chromatographic peaks with distinct shapes are identified as 1.5–4.5 min and 10–15 min, respectively. A magnified comparison of these peaks within both retention time intervals (1.5–4.5 min and 10–15 min) is presented. A comparison of the two figures demonstrated that the peak height of DR/SBSA was significantly lower than that of the unsulfurized group. The reduced peak height was correlated with lower VOCs emissions in DR/SBSA compared to UDR/SBSA.

#### 3.2.2. VOCs Components of SBSA

According to Section 3.2.1, the composition of SBSA is shown in Table 5.

As shown in Table 5, VOCs are produced by SBSA through simultaneous pyrolysis and oxidative degradation processes when subjected to elevated temperatures. The SBSA is designated as the control group, in which the SBS is incorporated. These components are primarily derived from the pyrolytic degradation of both the base asphalt and the SBS modifier. Three predominant classes of volatile small molecules were identified in the compound components: low-carbon alkanes represented by n-butane, oxygenated alcohols exemplified by 2,3-dimethyl-1-butanol, and carbonyl compounds dominated by hexanal. The compositional profile exhibited exceptionally clean characteristics and maintained structural integrity under thermal stress. The relative peak area of 2,4,6-tri-tert-butylphenol significantly exceeded those of other products, which is predominantly attributed to its superior thermal resistance. This compound is generated through radical recombination between benzene rings, originating from SBS pyrolysis and migrating tert-butyl groups during thermal decomposition. The stability of the molecule was significantly enhanced by the introduction of tert-butyl groups, which rendered 2,4,6-tri-tert-butylphenol resistant to decomposition or transformation during pyrolysis. As a result, it constitutes the dominant fraction in the product distribution. As evidenced by Table 4, Table 5 and Table 6, a spectrum of oxygenated and aromatic compounds is identified in both DRA and UDRA, demonstrating that rubber powder incorporation alters the VOCs profile through thermal cracking and oxidative aging pathways.

#### 3.2.3. VOCs Common Components of DR/SBSA and UDR/SBSA

**Figure 5:**
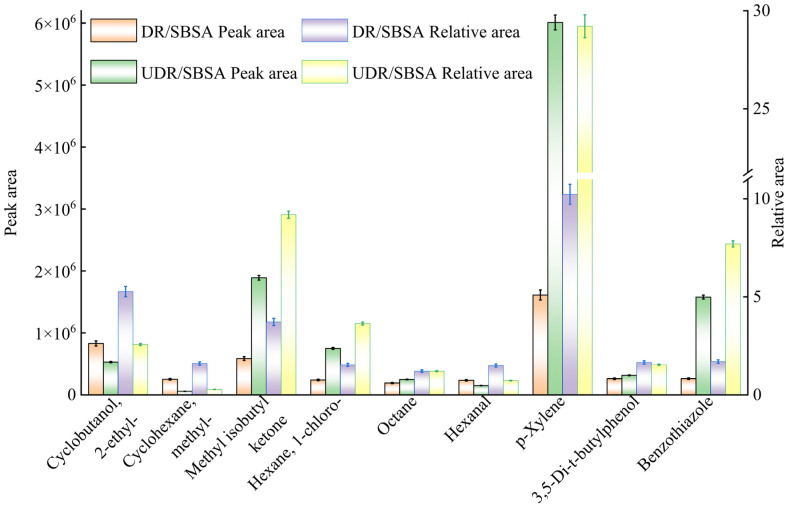
Peak area/relative area of compounds common to DRA and UDRA.

The common components identified in DR/SBSA, UDR/SBSA, and SBSA are listed in Table 6. In Table 6, the common compounds identified in SBS-modified asphalt before and after desulfurization are presented through qualitative analysis. Figure 5 illustrates the peak area/relative area of these common compounds. In chromatographic analysis, the absolute peak area reflects the absolute content of a compound within the sample, which is determined through mathematical integration of the area beneath the chromatographic peak performed by analytical software. Conversely, the relative peak area represents the proportional contribution of a specific compound to the total detectable components, obtained by dividing its individual peak area by the aggregate peak area of all components and multiplying the result by 100%.

From Table 6, these substances are characterized by relatively simple structures and were presented at low concentrations in the samples. Although some exhibit volatility or photochemical reactivity, the overall environmental toxicity and persistence risks are generally well controlled. As a result, they remain stable base emission components in SBS-modified systems.

Compared to SBSA, the introduction of rubber powder significantly alters the composition of VOCs. Several structurally more complex compounds were introduced, some of which may pose potential environmental risks. This change shows that rubber powder not only enhances asphalt performance but also introduces a new source of VOCs. Against this backdrop, a further comparison of VOCs released from rubber powder/SBS before and after desulfurization reveals the intrinsic relationship between rubber powder desulfurization and VOCs environmental risks.

As shown in Table 7, at the same 15% rubber powder content, more complex and toxic components were detected in the DR. Zhou et al. [17] pointed out that in volatile organic compound emissions, o-xylene and benzene are the most harmful, specifically represented as o-xylene and benzothiazole in Table 7. From Figure 5, it is evident that both p-xylene and benzothiazole exhibit significantly higher relative and absolute peak areas compared to other VOCs among the detected harmful substances. Based on these observations, these two VOCs were selected for further analysis.

Benzothiazole derivatives are recognized as a class of biologically active compounds. Multiple pharmacological effects have been documented, including antifungal activity, insecticidal efficacy, and nematicidal properties. Additionally, acaricidal, antiviral, and herbicidal functions are exhibited by these compounds. Their plant growth regulation capability has been widely reported, with characteristic advantages of low toxicity and high efficiency being consistently observed in experimental studies [36]. As shown in Figure 5, benzothiazoles were detected in both types of SBS-modified asphalt containing waste tire rubber powder, with a relative area not exceeding 10%. Benzothiazole compounds are conventionally employed as vulcanization accelerators in the production of waste tire rubber powder. Their residues are liberated during the high-temperature preparation of modified asphalt. Additionally, the relative area of benzothiazole in DR/SBSA was observed to decrease by 78% compared to UDR/SBSA, with only 1.7% remaining. This indicates that the generation and release of benzothiazole can be effectively regulated by DR, ensuring environmental safety.

Xylene, as a benzene-containing compound, is classified by its lipophilicity, toxicity, and persistence. These properties enable significant environmental accumulation to be observed, with bioaccumulation potential being well documented in environmental chemistry studies [37]. Exposure to xylene can induce neurotoxic effects, causing substantial damage to brain tissue. Xylene may also induce reproductive toxicity, developmental toxicity in embryos, and respiratory toxicity [38]. From Figure 5, it can be seen that the relative area of xylene is measured to be 19% higher in UDRA than in DRA, accounting for 29.2% of the total composition and being identified as the main component of UDRA. UDR is primarily derived from waste tires, with the rubber powder not subjected to desulfurization, retaining abundant disulfide bonds and sulfur-containing additives. These components can be released as reactive sulfur compounds under high-temperature processing, promoting the dehydrogenation of alkyl/cycloalkyl structures in asphalt and subsequent aromatization, which leads to the generation of aromatic compounds including xylene.

Moreover, the poor thermal cracking stability of the original rubber matrix in this rubber powder results in easier fragmentation into low-molecular-weight aromatic hydrocarbons during mixing, accompanied by increased accumulation of volatile organic compounds. Furthermore, the limited interfacial compatibility between UDRA and asphalt/SBS causes local accumulation of light aromatic components in the system, while their diffusion and release capabilities are reduced.

In contrast, when DR is utilized for SBS-modified asphalt production, the disruption of the original sulfur cross-linking structure, combined with enhanced thermal stability and dispersion capability, effectively suppresses the generation and retention of aromatic hydrocarbons.

#### 3.2.4. VOCs Unique Components of DR/SBSA and UDR/SBSA

Table 8 and Table 9 present the unique compound data for DRA and UDRA, respectively.

As indicated in Table 8 and Table 9, at a rubber powder content of 15%, the characteristic compound of DRA is identified as 3,5-di-tert-butylphenol, whereas that of UDRA is determined to be 2,5-di-tert-butylphenol. These phenolic compounds are suggested to originate from SBS-modified asphalt, which has been reported to release significant amounts of 2,4,6-tri-tert-butylphenol at 15.43 min.

In UDR, the retention of a dense cross-linked network and rubber segments within its structure may result in physical adsorption or embedding of large molecular phenolic compounds, thereby limiting their release within the system. Through desulfurization treatment, the original cross-linked structure is weakened, and more polar sites are exposed, facilitating a more complete release of residual components in SBS. Consequently, higher peak levels of compounds analogous to 3,5-di-tert-butylphenol are observed in this system. The presence or absence of desulfurization in rubber powder has been found to significantly influence the VOCs released from asphalt after heating. UDR is prone to intense decomposition reactions under high temperatures, and its sulfur-containing components are observed to exhibit catalytic tendencies toward aromatization, thereby promoting the formation of medium-to-high molecular weight oxidation–cracking products such as cyclohexanone and tetradecane. These substances, characterized by high volatility and environmental persistence, are recognized as ozone precursors that can be accumulated in ecological cycles, leading to air odor pollution and subsequent migration into water or soil systems, which poses long-term ecological risks.

Following desulfurization treatment, the surface polarity of rubber powder is enhanced, causing VOCs generated during heating to preferentially form low-toxicity, readily degradable small molecules such as branched alkanes and alcohols. This modification facilitates the timely release and dispersion of volatile compounds, effectively mitigating local aggregation phenomena.

### 3.3. VOCs Emission Mechanism

#### 3.3.1. FTIR Analysis

**Figure 6:**
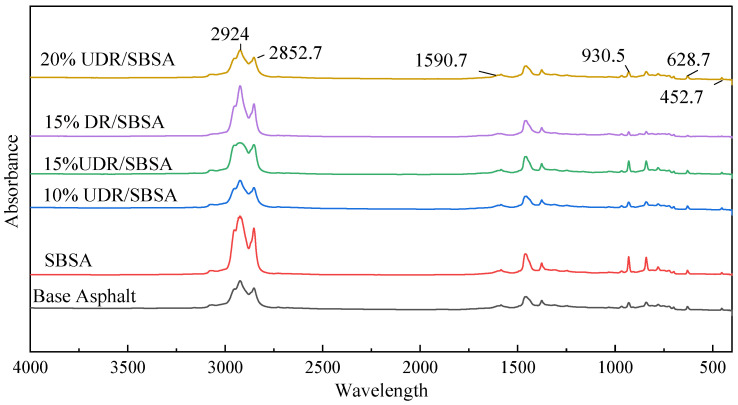
Comparison of infrared spectra of various asphalts.

FTIR can rapidly and accurately analyze the chemical bond characteristics and functional group information of substances, making it suitable for both qualitative and quantitative analysis. To further validate the results discussed above, infrared spectroscopy was used to verify the changes in key functional groups, as shown in Figure 6.

As shown in Figure 6, the peaks at 2924 cm^−1^ and 2852.7 cm^−1^ are attributed to C–H stretching vibrations in the alkane groups, with their changes reflecting the structure and interfacial properties of the polymer powder. In the UDRA system, these two peaks are strong, indicating that the cross-linked network of the gel powder remains intact, with tightly packed molecular chains. This structure restricts VOCs release and may lead to retention and sudden release. In contrast, in the DRA system, the peaks are weakened or broadened, indicating that the cross-linked structure has been disrupted by desulfurization, causing the molecular chains to loosen. This results in improved dispersion and interfacial reactivity, facilitating the smooth release of VOCs and reducing emission risks.

A significant absorption peak is observed at 1590.7 cm^−1^ in UDR, attributed to the stretching vibration of aromatic C=C bonds, primarily associated with the para-xylene structure. This indicates a high content of aromatic compounds. This characteristic is consistent with the GC-MS results, and the spectrum also shows higher levels of aromatic hydrocarbons, such as para-xylene, in the unsulfurized rubber powder. These findings confirm that sulfur plays a catalytic role in the transformation of non-aromatic components into aromatic ones.

A weak vibration absorption peak at 628.7 cm^−1^, corresponding to C–S bonds, was observed, with its intensity in DR significantly lower than in UDR, further supporting the GC-MS results, which show that sulfur-containing compounds, such as benzothiazole, are significantly lower in desulfurized rubber powder compared to UDR. The absorption peak at 930.5 cm^−1^ corresponds to the vibration of sulfate esters (S–O), indicating the presence of sulfur oxides or aromatic sulfur-containing structures. The intensity in DR is also significantly lower than in UDR. Furthermore, the absorption intensity at 1590.7 cm^−1^ in the desulfurized rubber powder decreases significantly, indicating a reduction in aromatization, which is linked to the decreased sulfur catalytic activity caused by desulfurization. In addition, the absorption peak at 628.7 cm^−1^ is almost completely absent, possibly due to the low content of sulfur-containing compounds, such as benzothiazole, in the desulfurized rubber powder, making them difficult to detect by the instrument. Additionally, new absorption peaks appear in the 1200–1150 cm^−1^ range, likely associated with phenolic hydroxyl (O–H) vibrations. Based on GC-MS detection results, these peaks are likely attributed to phenolic derivatives generated during desulfurization, such as 3,5-di-tert-butylphenol.

Cross-validation was performed using the results from headspace GC-MS and FTIR analysis. The aromatic content detected by GC-MS is directly proportional to the intensity of the 1590.7 cm^−1^ peak in the FTIR spectrum, indicating that aromatic hydrocarbon formation is influenced by sulfur, with UDR producing higher levels of p-xylene. Benzothiazole was detected only in the unsulfurized sample, corresponding to a weak peak at 628.7 cm^−1^. Additionally, the characteristic S-S bond peak in the 450–500 cm^−1^ range was observed exclusively in UDR, indicating the presence of sulfur-containing structures.

#### 3.3.2. SEM Analysis

**Figure 7:**
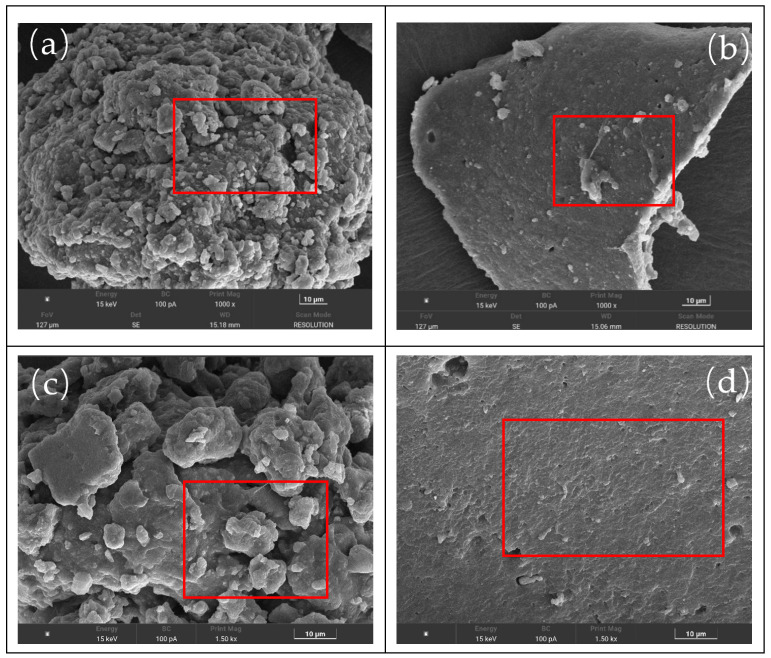
SEM images of modified asphalt at different magnifications: (**a**) UDR at 1000×; (**b**) DR at 1000×; (**c**) UDR at 1500×; (**d**) DR at 1500×.

**Figure 8:**
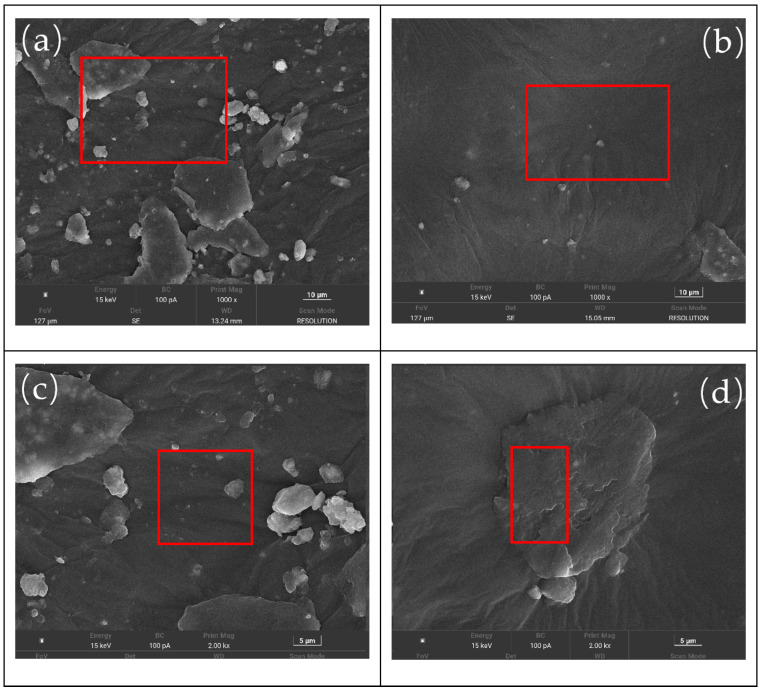
SEM images of modified asphalt at different magnifications: (**a**) UDR/SBSA at 1000×; (**b**) DR/SBSA at 1000×; (**c**) UDR/SBSA at 1500×; (**d**) DR/SBSA at 1500×.

SEM can reveal the microstructure of rubber in asphalt, providing further insights into its volatile VOCs emissions. The bonding between asphalt and rubber, the surface morphology of rubber particles, and their dispersion in asphalt all significantly influence VOCs volatilization and release. Figure 7 shows the SEM images of rubber powder particles, and Figure 8 shows the SEM images of the rubber powder-/SBS-modified asphalt.

##### The SEM Images of Rubber Powder Particles

From Figure 7, the images reveal that UDR particles exhibit a distinct agglomerated state, with significant variations in particle size, a multi-faceted and rough surface, and numerous small particles. Although some pores are observed locally, the overall structure is dense and lacks effective diffusion channels. This morphological characteristic suggests that the internal cross-linked network remains relatively intact, with rubber chains tightly entangled and particles firmly bound together, resulting in poor dispersion. The structural density restricts the migration and release pathways of organic small molecules, causing VOCs precursors to accumulate more easily within the particles. As the system temperature increases, these accumulated components are often released in a sudden burst, leading to high VOCs release rates and increased pollution risks.

In contrast, the DR particles shown in Figure 7b exhibit clearer contours, a smoother surface, significantly reduced attachments and pores, and a more regular overall structure with improved dispersion. The change results from the breaking of certain cross-linking bonds during the desulfurization process. It loosens the rubber molecular chains, reduces interparticle bonding strength, and creates a microstructural environment that promotes gas diffusion. These structural characteristics enable VOCs to be gradually released during the initial heating phase, preventing concentrated accumulation and explosive release at high temperatures, thereby improving control over both emission intensity and release rate.

High magnification Figure 7c,d further support the above conclusions. UDR particles exhibit rough, irregular surfaces, are tightly interlocked, and are coated with a large amount of gel-like residues and fine particles. Local cracking and edge curling are visible, reflecting a highly cross-linked three-dimensional network structure. This structure not only inhibits interface interactions between the rubber powder and the external environment but also obstructs the migration and release of internal VOCs. In contrast, DR samples exhibit a more compact and smooth particle surface with clear boundaries and uniform particle distribution, with little to no agglomeration. Due to their more open internal structure and enhanced chain segment mobility, VOCs diffuse and release smoothly during heating, effectively alleviating local enrichment and reducing emission peaks during the asphalt construction phase.

##### The SEM Images of the Rubber Powder/SBS-Modified Asphalt

As shown in Figure 8a, UDR/SBS particles predominantly exist as independent particles in the matrix. Although some particles exhibit slight swelling, no significant fusion occurs between particles, and they do not form a continuous swollen structure with SBS or asphalt. The interface bonding is also relatively weak. This structure suggests the internal cross-linking network remains largely intact, leading to poor mobility of rubber chains, low particle activity, and limited interfacial reactions, making effective synergy with the matrix difficult. The closed nature of this structure causes VOCs to accumulate within the particles and limits release pathways. This could lead to delayed release or transient concentration peaks during the construction heating phase, thereby increasing emission risks.

In contrast, the DRA particles in Figure 8b are more uniformly distributed, exhibiting regular morphology and consistent particle size with almost no large unbroken particles. The interface between the particles and asphalt transitions more smoothly. The desulfurization process disrupts the original cross-linked network of the rubber powder, making the molecular chains more flexible and deformable. This allows them to better integrate into the SBS network and asphalt matrix, enhancing compatibility and synergistic effects among the three components. The rubber powder particles are distributed throughout the matrix like rivets and enhance structural stability by providing smoother pathways for VOCs diffusion and release. This facilitates the gradual release of organic compounds during the early heating stage, effectively preventing their accumulation and sudden release at high temperatures.

Figure 8c,d show images at higher magnifications, further revealing the microscopic interface differences between the two systems. In Figure 8c, the UDR surface is rough and irregular, with noticeable agglomeration between particles and clear boundaries with the surrounding asphalt, showing almost no fusion. No signs of synergistic deformation between particles are observed, indicating that the cross-linked structure remains relatively dense, limiting molecular-level diffusion and reactions, which hinders VOCs release and emission control.

In Figure 8d, the interface transition between DR and asphalt is more natural, showing noticeable delamination on the particle surfaces and indicating that the internal structure has loosened. The particle edges exhibit a ‘radial’ extension towards the matrix, forming a multi-point contact morphology. This structure enhances interface bonding and facilitates synergistic deformation between the rubber powder and SBS. More importantly, the more open and flexible interface structure provides pathways for the timely release of VOCs, allowing gradual diffusion or reaction with the matrix during heating, reducing stagnation and accumulation, and lowering both emission intensity and overall emission load per unit time.

## 4. Conclusions

To reduce VOCs emissions from rubber powder/SBS-modified asphalt under high temperatures, the key road performance characteristics of asphalt modified with different proportions of rubber powder/SBS were investigated. The optimal rubber powder proportion was determined. The VOCs emission behavior of rubber powder/SBS-modified asphalt before and after desulfurization was compared and analyzed using GC-MS. The VOCs emission mechanisms were revealed through microstructural testing. This expands the utilization rate of waste rubber powder and reduces its environmental pollution. This improves the service life and environmental friendliness of asphalt pavement throughout its entire lifecycle, contributing to achieving sustainable development goals. The research results are as follows:

(1) As the rubber powder content increases, the softening point of DR/SBSA and UDR/SBSA initially increases and then decreases, with the best high-temperature performance observed at a rubber powder content of 15%. At 15% rubber powder content, the ductility of DR/SBSA reaches 48.6 cm. This value is 9% higher than that of UDR/SBSA at the same content. It is also 14% higher than DR/SBSA with other contents. The softening point of DR/SBSA is 76.3 °C. This is 4% higher than that of UDR/SBSA (73.2 °C). After aging, the rutting factor of 15% DR/SBSA increases by 24% compared to the unaged state. Under the same conditions, UDR/SBSA shows a 33% increase. Therefore, DR/SBSA with 15% content exhibits the best overall performance.
(2) GC-MS analysis indicates that at a rubber powder content of 15%, UDR/SBSA exhibits more complex and toxic components, particularly diphenylmethane. The relative area of UDR/SBSA is 19% higher than that of DR/SBSA. Additionally, diphenylmethane accounts for 29.2% of the relative area, making it the primary VOCs component in UDR/SBSA. As the rubber powder content increases, the concentration of released VOCs also increases.
(3) Infrared spectroscopy can assist in verifying the GC-MS analysis through functional group changes, such as C–H, C=C, C–S, S–O, and S–S. These spectral data confirm that the sulfur content in desulfurized rubber powder is extremely low, reducing VOCs emissions.
(4) SEM observations revealed that the surface of desulfurized rubber powder is more compact and regular, with uniform particle distribution and significantly reduced pore size, exhibiting a closed and continuous structure. Desulfurization treatment disrupts part of the cross-linked network, enhancing the dispersion and interface adaptability of rubber powder in asphalt, facilitating the formation of a stable and uniform modified system. This improves the overall performance of the material and reduces VOCs emissions.
(5) This study focused on the VOCs emission characteristics and optimal rubber powder content of devulcanized rubber powder/SBS-modified asphalt, as well as its related road performance. However, it did not include its application in actual asphalt mixtures. Future work should apply it to asphalt mixtures and test their performance, such as fatigue and aging resistance.

## Figures and Tables

**Table 1:** Main technical indicators of base asphalt.

Test Item		Test Value	Technical Specifications
Needle Penetration (25 °C, 0.1 mm)		68	60–80
Softening Point (global method/°C)		48	≥46
Ductility (15 °C/cm)		>100	≥100
Rtfo (163 °C/85 min)	Quality Loss/%	0.4	≤±0.8
	Ductility (10 °C/cm)	12	≥4
	Needle Penetration (25 °C, 0.1 mm)	60	≥58

**Table 2:** Main technical indicators of desulfurization powder.

Test Items	Test Results	Technical Indicators	Test Methods
Relative Density	1.17	1.10~1.30	ASTM D6114 [29]
Moisture Content	0.9	<1.0%	ASTM D5603 [30]
Metal Content	0.023	<0.05%	ASTM D6114
Rubber Hydrocarbon Content	48	≥42%	ASTM D5755 [31]
Mooney Viscosity	33	≤40%	ASTM D5755
Carbon Black Content	29	≥24%	ASTM D5755
Ash	7	≤8%	ASTM D5755
Acetone Extract	14	≤20%	ASTM D5755

**Table 3:** Main technical specifications of SBS modifiers.

Test Items	Inspection Data
Structure	Linear
Tensile Strength	20 (Mpa)
Shore Hardness	76
Melt Flow Rate (MFR)	0.1 (g/10 min)
Viscosity of 25% Toluene Solution at 25 °C	2455 (Mpa·s)

**Table 4:** Test Types.

Asphalt Type	Modifier Dosage	Test							
		Penetration	Softening Point	Ductility	Rolling thin-film oven	Rheological Properties	Gas chromatography–mass spectrometry	Fourier Transform Infrared Spectroscopy	Scanning Electron Microscopy
SBSA	4%SBS	√	√	√		√	√	√	
DR/SBSA	4%SBS + 10%DR	√	√	√		√			
	4%SBS + 15%DR	√	√	√	√	√	√	√	√
	4%SBS + 20%DR	√	√	√		√			
UDR/SBSA	4%SBS + 10%UDR	√	√	√		√	√	√	
	4%SBS + 15%UDR	√	√	√	√	√	√	√	√
	4%SBS + 20%UDR	√	√	√		√	√	√	

**Table 5:** Information on SBSA components.

No.	Name	CAS	Retention Time (min)	Matching Degree (%)	Molecular Formula	Peak Area	Relative Area
1	2,3-Dimethyl-1-butanol	19550-30-2	1.51	87	C_6_H_14_O	156383.3	1.79
2	2-Ethylcyclobutanol	35301-43-0	1.59	87	C_6_H_12_O	424618.21	4.87
3	4-Chloro-1-octene	999-07-5	1.87	85	C_8_H_17_Cl	267003.04	3.06
4	3,7-Dimethyl-1-octene	4984-01-4	1.99	86	C_10_H_20_	109020.43	1.25
5	Methylcyclohexane	108-87-2	2.29	81	C_7_H_14_	37255.77	0.43
6	1-Chlorohexane	544-10-5	2.72	91	C_6_H_13_Cl	141674.85	1.63
7	1-Octene	111-65-9	3.08	90	C_8_H_18_	145928.9	1.67
8	1-Hexanol	66-25-1	3.14	91	C_6_H_12_O	151444.09	1.74
9	4-(1,1-Dimethylethyl)phenol (BHT)	80-46-6	13.03	87	C_11_H_16_O	435440.93	5
10	2,4,6-Tri-tert-butylphenol	732-26-3	15.43	96	C_18_H_30_O	4829724.49	55.43

**Table 6:** Common Components of DR/SBSA, UDR/SBSA, and SBSA.

No.	Name	CAS	Retention Time (min)	Matching Degree (%)	Molecular Formula
1	Cyclobutanol, 2-ethyl-	35301-43-0	1.59	85	C_6_H_12_O
2	Cyclohexane, methyl-	108-87-2	2.3	87	C_7_H_14_
3	Hexane, 1-chloro-	544-10-5	2.71	91	C_6_H_13_Cl
4	1-Octene	111-65-9	3.07	92	C_8_H_18_
5	1-Hexanol	66-25-1	3.14	90	C_6_H_12_O

**Table 7:** Information on Compounds Common to DR/SBSA and UDR/SBSA.

No.	Name	CAS	Retention (min)	Matching Degree (%)	Molecular Formula
1	2-Ethylcyclobutanol	35301-43-0	1.59	85	C_6_H_12_O
2	Methylcyclohexane	108-87-2	2.3	87	C_7_H_14_
3	Methyl isobutyl ketone	108-10-1	2.38	87	C_6_H_12_O
4	1-Chlorohexane	544-10-5	2.71	91	C_6_H_13_Cl
5	1-Octene	111-65-9	3.07	92	C_8_H_18_
6	1-Hexanol	66-25-1	3.14	90	C_6_H_12_O
7	p-Xylene	106-42-3	4.33	97	C_8_H_10_
8	Benzothiazole	95-16-9	10.7	88	C_7_H_5_NS
9	3,5-bis(1,1-dimethylethyl)phenol	26886-05-5	14.43	88	C_12_H_18_O

**Table 8:** Information on Compounds Unique to DRA.

No.	Name	CAS	Retention Time (min)	Matching Degree (%)	Molecular Formula
1	3-Methylpent-4-en-1-ol	51174-44-8	2	86	C_6_H_12_O
2	4-Propylheptane	3178-29-8	2.6	82	C_10_H_22_
3	3,5-Di-tert-butylphenol	1138-52-9	14.4	92	C_14_H_22_O

**Table 9:** Information on Compounds Specific to UDRA.

No.	Name	CAS	Retention Time (min)	Matching Degree (%)	Molecular Formula
1	Cyclohexanone	1138-52-9	4.8	94	C_14_H_22_O
2	Tetradecane	629-59-4	10.1	87	C_14_H_30_
3	2,5-Di-tert-butylphenol	5875-45-6	14.4	92	C_14_H_22_O

## Data Availability

The original contributions presented in this study are included in the article. Further inquiries can be directed to the corresponding author.
